# Demographic rates reveal the benefits of protected areas in a long-lived migratory bird

**DOI:** 10.1073/pnas.2212035120

**Published:** 2023-03-13

**Authors:** Andrea Soriano-Redondo, Richard Inger, Richard B. Sherley, Eileen C. Rees, Fitsum Abadi, Graham McElwaine, Kendrew Colhoun, Olafur Einarsson, Sverrir Thorstensen, Julia Newth, Kane Brides, David J. Hodgson, Stuart Bearhop

**Affiliations:** ^a^Centre for Ecology and Conservation, University of Exeter, Penryn, Cornwall TR10 9FE, UK; ^b^Helsinki Lab of Interdisciplinary Conservation Science, Department of Geosciences and Geography, University of Helsinki, 00014 Helsinki, Finland; ^c^Wildfowl and Wetlands Trust, Slimbridge, Gloucestershire GL2 7BT, UK; ^d^Department of Zoology, University of Cambridge, Cambridge CB2 3EJ, UK; ^e^Department of Fish, Wildlife and Conservation Ecology, New Mexico State University, Las Cruces, NM 88003; ^f^100 Strangford Road, Downpatrick, County Down BT30 6LZ, UK; ^g^KRC Ecological Ltd., Bryansford, County Down BT33 0PZ, UK; ^h^Smàrarima 39, IS-112 Reykjavik, Iceland; ^i^Langahlíð 9a, IS-603 Akureyri, Iceland

**Keywords:** protected area, demography, migration

## Abstract

Recent studies have suggested that protected areas often fail to conserve target species. However, the efficacy of terrestrial protected areas is difficult to measure, especially for highly vagile species like migratory birds that may move between protected and unprotected areas throughout their lives. Here, we use a 30-y dataset of detailed demographic data from a migratory waterbird, the Whooper swan (*Cygnus cygnus*), to assess the value of nature reserves (NRs). We assess how demographic rates vary at sites with varying levels of protection and how they are influenced by movements between sites. Swans had a lower breeding probability when wintering inside NRs than outside but better survival for all age classes, generating a 30-fold higher annual growth rate within NRs. There was also a net movement of individuals from NRs to non-NRs. By combining these demographic rates and estimates of movement (into and out of NRs) into population projection models, we show that the NRs should help to double the population of swans wintering in the United Kingdom by 2030. These results highlight the major effect that spatial management can have on species conservation, even when the areas protected are relatively small and only used during short periods of the life cycle.

## Significance

The conservation of the natural world currently relies on the establishment of protected areas. However, site protection alone does not guarantee good biodiversity outcomes. Here, we take advantage of a 30-y dataset on Whooper swans which provides a rare opportunity to quantify the role of nature reserves in the population dynamics of a migratory waterbird. We find that nature reserves play a key role by boosting the survival of this species and will effectively double its population size by 2030.

Global biodiversity is undergoing unprecedented declines (1, 2), with the designation of protected areas (PAs) being the most widely used strategy in attempts to slow and ultimately reverse these declines. Currently, PAs account for 16.6% of global land surface area and 7.7% of the ocean, which corresponds to 50.1 million km^2^ of terrestrial and aquatic ecosystems protected (3). The main goals of PAs are to maintain species richness and population abundances, and in turn preserve ecosystem function and deliver ecosystem services (4, 5). PAs can successfully maintain (6) and even boost populations: spillover effects have been reported from marine systems, where populations in surrounding areas are supplemented by individuals dispersing from PAs (7, 8). However, declines in abundance and species richness within PAs are not uncommon (9, 10). Indeed, a recent global study of waterbird population trends concluded that site protection alone did not guarantee good biodiversity outcomes (11).

For species that are highly vagile, PAs generally fail to cover sufficient geographic range to ensure their long-term protection (12, 13), leaving substantial numbers of individuals in unprotected areas where abundances are generally not monitored (14). Habitats and conditions are also likely to differ markedly between PAs and unprotected areas, and will often influence the demographic processes underlying abundances in very different ways (15–17). Hence, one of the reasons there might be such variation in the apparent success or failure of protected areas is that common measures of performance, such as population trajectories, might mask a more complex picture. Likewise, the true population trajectories and the demographic processes driving them may remain cryptic if we only focus on data from within PAs. To reveal the success or failure of PAs it is therefore critical to monitor abundances and the underlying demographic processes both within and outside of PAs in order to fully understand the population dynamics and implement subsequent conservation interventions where necessary. Given limited conservation budgets, however, this has rarely been achieved in practice.

In the case of migratory species, which are often more difficult to conserve (18), understanding the effectiveness of PAs is further complicated as multiple interdependent sites can be occupied across different life stages or seasons (19), with varying degrees of protection, and with considerable movement of individuals between them. In addition, the habitat and conditions experienced during one stage of the lifecycle may be carried over to influence the success of individuals in the following stage (20). For example, in migratory geese, conditions experienced during winter can affect breeding success in the subsequent breeding season (21), making the assessment of population change and its associate drivers even more difficult.

Finally, protected areas also vary widely in the amount of protection they actually afford, ranging from basic habitat protection to active species management (22). Here, we focus on nature reserves (NRs) as areas designed and managed for the conservation of target species and habitats, in contrast to other types of PA that offer less targeted protection.

Our case study comprises a unique 30-y dataset on a migratory waterbird, the Whooper swan (*Cygnus cygnus*), providing a rare opportunity to quantify the detailed and cryptic demographic parameters underpinning population dynamics both within and outside of NRs. Whooper swans breed in Iceland before migrating to the United Kingdom and Ireland where they spend the winter. Being large, conspicuous animals, they are highly amenable to counting and their numbers have been monitored to some degree on their wintering grounds for over 100 y (23), being recorded at over 2,000 sites, currently including 22 sites of international importance (each holding at least 340 individuals) (24). Of these sites, three relatively small areas (at Caerlaverock, Martin Mere, & Welney) are managed as NRs by the Wildfowl and Wetlands Trust (WWT), providing targeted protection for this species. Outside of these NRs, birds use a wide range of sites with varying levels of protection. Since 1980, a program for marking the birds with unique identification tags and an intensive resighting program have been carried out, both inside and out of NRs (25). The resulting individual-based dataset, comprising almost 223,000 observations of over 10,000 marked individuals, allows us to produce models of both survival and reproductive success and assess how these vital rates associate with a suite of predictors. We evaluate the effectiveness of NRs using a Bayesian framework which allows us to combine estimates of survival and reproduction, and their associated uncertainties, into demographic models that capture both vital rates inside and outside of NRs and rates of movement into and out of NRs. Finally, we use these models to predict future population trajectories both with and without the presence of NRs.

## Results

### Survival Models.

**Fig. 1.**
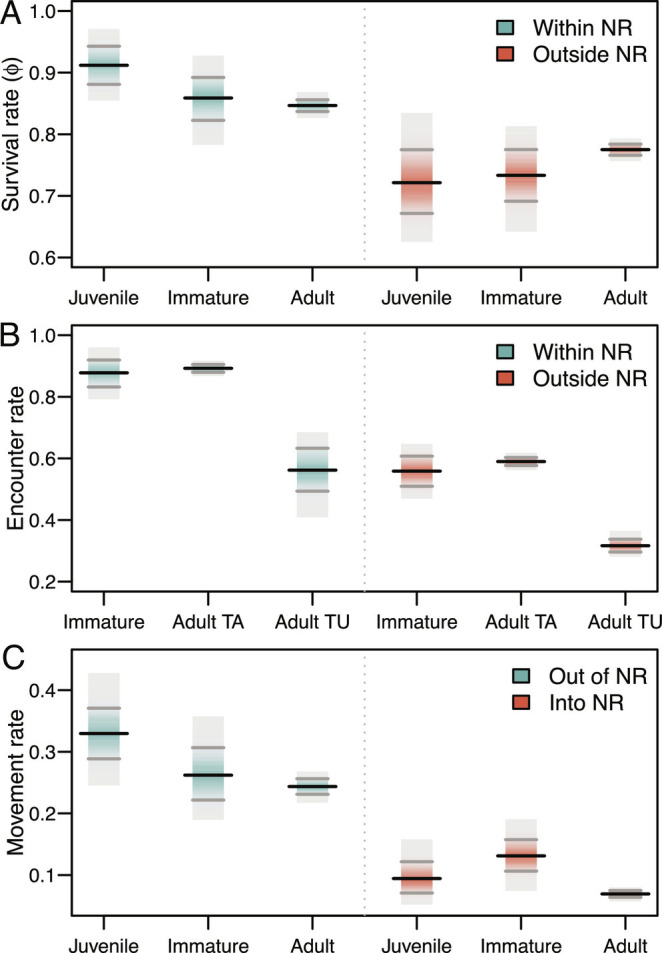
Whooper swans’ (*A*) survival and (*B*) encounter rates within and outside nature reserves, and (*C*) movement rate out of and into NR. Note, to account for trap dependence, we considered different encounter probabilities for trap-aware adults (TA) and trap-unaware adults (TU). Black tick marks denote posterior means, gray tick marks denote 95% credible intervals and the extent of the gray polygons shows the extent of the posterior distribution.

In long-lived species, such as swans, survival is a key demographic parameter with a strong impact on population dynamics (26). Multiple observations of individually identifiable animals can be used to accurately estimate apparent survival. Where dispersal occurs, multistate capture-mark-recapture (CMR) methods can be used to incorporate movement probabilities into the estimation process by considering observations of marked individuals from multiple sites (27). We used a multistate CMR model, implemented in a Bayesian framework, and encounter (or resighting) histories for 6,952 individual swans to estimate survival ( ϕ ) of different age classes (juvenile, immature, and adult birds) within and outside of NRs (Fig. 1*A*), having accounted for age-dependent probabilities of encounter ( ρ ) and trap-awareness in adults (Fig. 1*B*) and their movement ( ψ ) probabilities between sites (Fig. 1*C*).

For all age classes, our multistate CMR model shows that mean (SD) survival was considerably higher inside NRs than outside of NRs: juveniles = 0.91 (0.02) vs. 0.72 (0.03); immatures = 0.86 (0.02) vs. 0.73 (0.02); and adults = 0.85 (0.004) vs. 0.77 (0.004) (Fig. 1*A*). Swans were more likely to move out of NRs rather than move into NRs between wintering seasons (Fig. 1*C*). Juveniles in NRs had the highest mean probability of transitioning out of NRs (0.33, SD = 0.02) followed by immature swans (0.26, SD = 0.02) and adult swans (0.24, SD = 0.01). Movement probabilities into NRs were highest for immature swans (0.13, SD = 0.01), followed by juveniles (0.09, SD = 0.01) and adults (0.07, SD = 0.003).

### Productivity.

In addition to survival and movement, the other key determinant of demographic change is productivity. In Whooper swans, overall population breeding success (the number of offspring produced per breeding season) can be quantified because juveniles can be identified by scanning flocks of swans in fields (28). In addition, brood size can also be measured as swans maintain family groups during the winter. An understanding of the underlying processes that determine overall population-level breeding success, and how these change with time and with environmental conditions, however, can only be derived from long-term data on individually identifiable animals.

While reproduction takes place on the swan’s Icelandic breeding grounds, the body condition of the adults upon arrival to breed is largely determined by conditions experienced during the winter in the United Kingdom and Ireland, which subsequently carries over to affect the probability of breeding (20, 21, 29). To assess this carry-over effect on the probability of breeding, we constructed generalized linear mixed models with a binary code denoting whether an individual had successfully bred in a given year (1) or not (0) as the dependent variable (*Materials and Methods*). As predictors in the model, we used age, the quadratic term for age, a measure of individual breeding experience, and winter site use in the two winters before the observation of successful breading. Individuals that spent most of their time in NRs in the previous two winters were defined as wintering within NRs, while those that spent most of their time outside of NRs were defined as such. We also defined two other categories: those which spent the first winter within NRs then moved out in the second winter, and vice versa. Model selection was achieved in a frequentist framework due to the difficulties associated with model selection in a Bayesian framework (30, 31). The minimally adequate model was then constructed in a Bayesian framework to allow the uncertainties to be incorporated into subsequent demographic models.

**Fig. 2.**
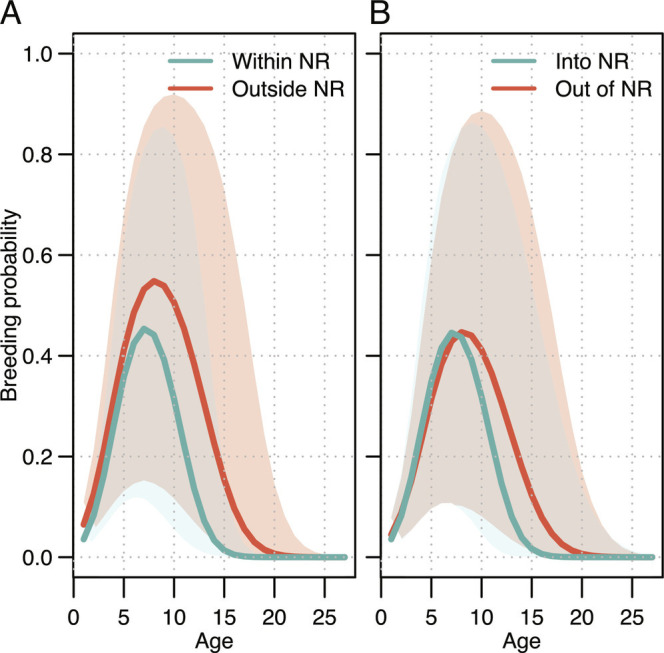
Whooper swans’ breeding probability with age for (*A*) individuals within and outside nature reserves (NR) during the two preceding winters, and (*B*) for individuals moving from outside nature reserves in the previous winter to inside nature reserves (into NR) and vice versa (out of NR). Solid lines denote the posterior mean and colored polygons the extent of the 95% credible intervals.

Swans wintering in NRs had a lower breeding probability than those wintering in unprotected areas (Fig. 2 and *SI Appendix*, Fig. S1 and Table S1). For all swans, mean (SD) breeding probabilities varied with age, peaking at 0.46 (0.20) at age seven for birds inside NRs and at 0.54 (0.22) at age eight for birds outside NRs. Breeding probabilities then declined as birds aged, although birds outside NRs maintained a higher breeding probability for longer than birds inside NRs (Fig. 2*A*); for example, the mean falls below 0.2 at 12 y old for birds in NRs, compared with 16 y for those outside of NRs. For birds that switched between NRs and non-NR sites, the maximum breeding probability was similar for birds using NRs, peaking at 0.46 (0.22) at age eight for birds moving out of NRs and 0.46 (0.21) at age seven for birds moving into NRs. Birds moving out of NRs did, however, benefit from higher probabilities of breeding over a greater number of years than birds inside NRs (Fig. 2 and *SI Appendix*, Fig. S1), with their mean probability only falling below 0.2 at 15 y old (Fig. 2*B*).

While the mechanisms behind these differences are largely beyond the scope of the current study, we identified two candidates for which we had data: density dependence (which can subsequently affect body condition), and the timing of departure from the wintering sites, both of which have the potential to influence productivity (32–34). We constructed a separate model to explore any potential effects of density dependence on individuals wintering at NRs using maximum bird counts at NRs as a proxy for density and found no significant effect of density on productivity (*SI Appendix*, Table S2*C*). Departure dates published for individual swans fitted with GPS tags in three separate years were summarized, to describe any differences in the timing of departure from within and outside NRs (35, 36). We found no significant differences in either departure from the main winter site, departure from the United Kingdom or Ireland, or arrival in Iceland between swans wintering inside and outside of NRs (*SI Appendix*, Fig. S2).

**Fig. 3.**
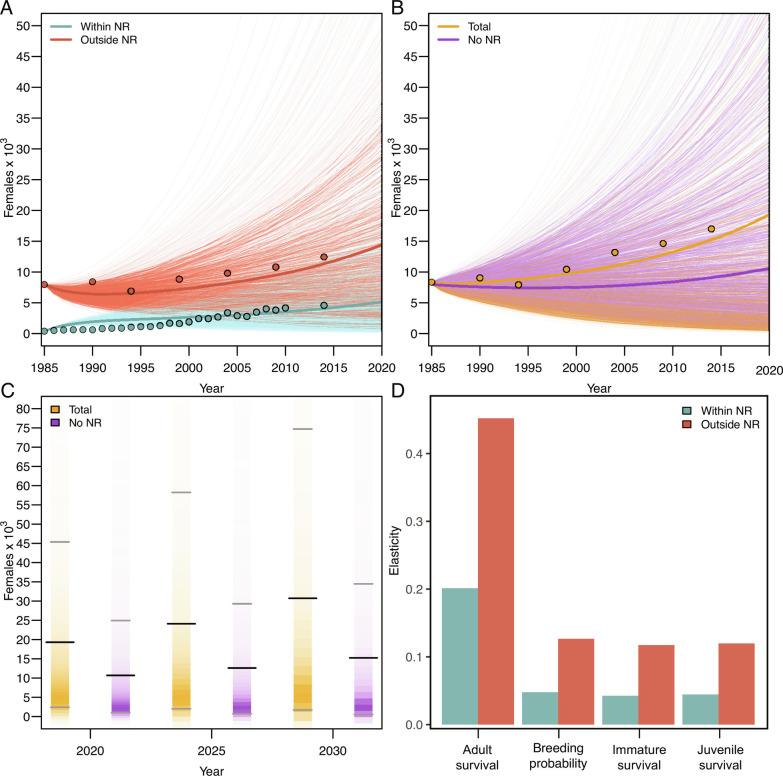
Bayesian population projection model with demographic stochasticity and parameter uncertainty to model the changes (*A*) within and outside nature reserves (NRs), and (*B*) in the total Whooper swan population size, and in a counterfactual scenario where NRs had never been set up. Dots represent actual population numbers from census data. Thick lines show the posterior mean and each thin line shows one iteration of the model. (*C*) Cross-sectional view of the demographic projections in (*B*) comparing the mean (±80% CIs, grey ticks) between the total population size and the scenario where NRs had never been set up, for the years 2020, 2025, and 2030. (*D*) Elasticity of the demographic parameters from the projections where swans were allowed to move between NRs and non-NRs.

At face value, these results paint a contrasting picture. Whooper swans that overwinter in NRs certainly survive better than their counterparts outside of NRs (Fig. 1*A*) and, considered in isolation, this would appear to be an obvious benefit of spatial protection. However, those same swans using NRs also tend to stop breeding earlier and breed with a lower probability each year throughout their lives (Fig. 2). Moreover, individual swans are almost three times as likely to leave the NRs as they are to move into them, with >30% of juvenile swans choosing not to return to NRs the following winter (Fig. 1*C*). Those juveniles that do leave then also suffer almost double the annual mortality (14% vs. 27%) in the subsequent year of life as immatures (Fig. 1*B*). Under certain conditions, high juvenile mortality can have important population-level consequences, even in long-lived vertebrates (e.g., ref. 37), and immature individuals are often important in the demographic process via compensatory recruitment (38). Finally, considering that, at best, the swans in NRs make up about 25% of the total population [(28); Fig. 3*A*]; it is clear that the complexity of the age-dependent effect makes it difficult to intuitively understand the population-level impact of NRs in this system. Accordingly, we constructed population models to identify differences in the population trajectories between swans utilizing NRs and non-NRs, also to understand how the overall population wintering in the British Isles might be altered by the removal of the NRs.

### Population Models.

Using the relevant posterior probability distributions, we parameterized stochastic projection models with 20 age-classes (1 juvenile, 1 immature, and 18 breeding adult classes) that incorporated our observed age-specific survival, breeding, and movement probabilities. Swans were able to breed between the ages of 2 and 20, and we ran two versions of the model: one where swans were allowed to transition between NRs and non-NRs (to simulate reality) and one where they were not, with the latter to simulate a counterfactual situation where the NRs had never been set up. We used population counts for 1985 to seed our model (28), projected until 2030, and compared our mean population trajectories to census data for the period 1985 to 2014 (39).

Population projections both within and outside the NRs increased in line with the respective observed counts (Fig. 3*A*). However, the mean (±SD) growth rate (λ) was faster for the population inside NRs (1.06 ± 0.03) than outside of NRs (1.002 ± 0.03), which was only growing at ~0.2% per year. The growth rate in the NRs was such that the population within NR went from ~5% of the total population in 1985 to ~27% in 2014, and our projections suggest that it will reach 50% by 2030 (Fig. 3). In short, our results suggest that the long-term benefit of these NRs will have been to double the effective population of Whooper swans wintering in the British Isles in the 45 y between 1985 and 2030 (Fig. 3 *B* and *C*).

Finally, elasticity estimates indicate that adult survival was the vital rate with the greatest influence on population growth, both within (0.20) and outside NRs (0.45), with similar patterns for breeding probability, immature survival, and juvenile survival (Fig. 3*D*). Population growth was most affected by perturbations occurring outside NRs, as these contain a higher proportion of the population during winter. Moreover, the ratio between the elasticity of adult survival and breeding probability was higher inside NRs (4.21) than outside (3.57). This indicates that adult survival took on a greater importance inside NRs, which is related to the higher adult survival and lower breeding probability experienced by individuals in these areas.

## Discussion

Protected areas are the main tool being used to stem declines in biodiversity (6), with a growing consensus that a global goal should be to designate 30% of the planet surface by 2030 (40). However, recent work, based on population trends, has suggested that protected areas may not always function in the way they are intended (11). Evidence from terrestrial and marine PAs indicate that the level of protection is an essential aspect in determining their effectiveness, with those under weak management or enforcement failing to increase populations of target species (7, 11, 41). Many populations utilize a variety of habitats with different levels of protection, from sites with targeted protection for specific species or groups to areas under more general protection, as well as unprotected ones. Despite this, there is little evidence for how demographic rates might vary under various levels of protection (16, 17, 42), and to our knowledge no previous empirical evidence on how demographic rates are influenced by movements between sites with different levels of protection.

Using a unique dataset combining data from both NRs with targeted protection for swans, and other sites affording varying or no protection, we found that Whooper swans wintering in NRs have higher survival but lower probability of breeding. Population models show that reduced breeding performance is more than compensated for by a longer lifespan. These demographic nuances illustrate how targeted protection within NRs can enhance population growth even when only a relatively small proportion of the population use these PAs within any given year.

Our results highlight that viewing population trends alone may mask the more complex dynamics that exist between PAs and the surrounding landscape. They also emphasize the need to measure demographic parameters both inside and outside of PAs when drawing conclusions about overall population dynamics. The annual population growth rate for the swans outside of the NRs was 0.2%, while inside NRs it was 6%—i.e., a 30-fold difference depending on the wintering sites used. Importantly, we were able to capture movement rates between NRs and non-NRs at different life stages, which highlighted a net loss of individuals, especially juvenile and immature birds, from NRs to areas often without specific protection. In this situation, undertaking counts only outside of the NRs would give a disproportionate sense of the real population growth rate in these areas. This is especially relevant in the context of source-sink dynamics, where populations in certain areas or habitats suffer net mortality and are supported demographically by immigration (43–45). We might expect PAs to often function as sources in this respect as they are commonly selected because they hold significant numbers of individuals. Dispersal movements between areas are difficult to detect without surveying multiple sites (and known individuals) over long time periods, thus highlighting the value of long-term, intensive monitoring programs.

In long-lived species, survival is the key parameter driving population growth, particularly the survival of adult individuals (26). Other studies have demonstrated the efficacy of PAs in increasing the survival of a wide range of taxa, such as leopards (*Panthera pardus*) (46) on land, and Hector’s dolphins (*Cephalorhynchus hectori*) (47) and European lobsters (*Homarus gammarus*) (48) in the marine environment. However, this issue has been largely unexplored for migratory animals that use multiple sites during their annual cycle, and for which the use of protected or unprotected areas during a relatively short interval of time could be considered trivial. Here, we demonstrate that PAs managed for the conservation of Whooper swans achieve the goal of reducing mortality in all age classes. This could be linked to the presence of fox-proof fences at WWT sites, supplementary food, managed roosting sites, the use of bird flight diverters to increase visibility of power lines, or the banning of hunting. Indeed, hunting can affect swans in two different ways. First, ringing recoveries data show that 13% of the individual marked birds reported as dead have been illegally hunted (25) and 13% of live individuals have been found to have lead shot embedded in their body tissues (49, 50). Second, ingestion of lead ammunition is a major source of poisoning in wild birds in the United Kingdom (51). Blood analyses in Whooper swans found that over 40% presented elevated lead levels, which affected body condition in severe cases (52). Thus, wintering in NRs could protect swans from the direct and indirect consequences of hunting.

More generally, the management actions targeted for Whooper swans are likely to benefit the larger group of waterbird species that overwinter in these NRs and the wetland habitats they use. Indeed, waterbirds play a fundamental role in many aquatic ecosystems, as predators, herbivores and seed vectors, and via pest control and nutrient cycling; thus, the increase in population numbers is likely to have positive cascading effects on the whole ecosystem (53).

Our results also show that birds wintering in NRs had lower reproductive success; breeding probability peaked sooner, at lower values, and decreased more quickly for birds wintering inside NRs than outside. For many migratory species, early arrival to the breeding grounds is key to being able to breed successfully (54, 55). However, individuals wintering inside and outside of NRs departed their wintering sites and arrived in Iceland on similar dates. Furthermore, even though density dependence can influence body condition and thus the timing of migration and breeding success, we did not find an association between the probability of breeding and the numbers of wintering individuals inside NRs. We cannot rule out an effect of density dependence since we could not test its effects outside NRs. An alternative explanation is that wintering in NRs yields a different life history strategy, whereby breeding probability is generally lower because of the larger residual reproductive value associated with a longer lifespan, as indicated by the different elasticity ratios between adult survival and breeding probability inside and outside NRs (56). It is also possible that the lower breeding probability within NRs occurs because NRs allow lower-quality, weak or ill individuals to survive that then cannot reproduce successfully in the subsequent breeding period, thus reducing the overall breeding probability for this group. This is, however, unlikely because there is no evidence of higher variance in breeding success among birds wintering in NRs (Fig. 2). Overall, birds wintering in NRs have more opportunities to reproduce throughout their lifespan and on average will achieve higher lifetime reproductive success.

Finally, the movement probabilities indicated a net movement of individuals away from NRs to non-NRs. Although at first glance this may appear problematic from a conservation standpoint, Whooper swans congregate in large numbers in NRs, which may drive density-dependent competition for resources, encouraging dispersal. Juvenile and immature birds would be less likely to compete successfully in such circumstances, and young animals are often still developing the strategies they will use in later life (57), both of which could explain the higher transition rates we saw in these age classes than in adults. These young individuals would recruit into the population outside the NRs and contribute to the population growth in these areas. This spillover effect that results from the dispersal of individuals from PAs to the surrounding areas is well studied in the case of marine reserves (7, 8), perhaps because of the potential to benefit fisheries (58), but it remains largely unexplored in terrestrial ecosystems (59, 60) (but see ref. 61).

In this study, we show how targeted management for the conservation of one species in three small areas, relative to both the wintering and annual range of the population, has had a major effect on its population dynamics, with projections showing that NRs will have effectively helped double the population by 2030. Trying to disentangle the relative contribution of each conservation measure (hunting ban, supplementary feeding, fox-proofed fencing, and power line mitigation) in the survival of this species would be key to prioritize the implementation of protection measures in other PAs. Moreover, implementing similar long-term monitoring programs for other waterbirds would allow us to better understand the impact of these NRs on the wider ecosystem.

Overall, our results highlight that many PAs will require much more detailed management plans for species of conservation interest in order to achieve positive outcomes. Perhaps most importantly, our study demonstrates that it is essential not to underestimate the contribution of localized protection for highly mobile species and that targeted measures during key periods of the life cycle can have disproportionate effects on the conservation of species.

## Materials and Methods

### Study Population.

The Icelandic-breeding population of Whooper swans, which is geographically isolated from other Whooper swan breeding populations, consisted of around 29,000 birds in 2005, increasing to 34,000 in 2015 (25, 62). Autumn migration to the wintering grounds occurs mainly in September and October, with the vast majority (approximately 94%) of birds spending November to March in the United Kingdom and the Republic of Ireland, before returning to the breeding grounds during March and April (25, 35). During both the spring and autumn migrations, birds make a sea crossing, which is probably the longest of any swan species without stopping at staging sites (63, 64).

### Marking and Resighting of Birds.

From 1980 onward, swans have been caught each year during the wintering period in decoy-type “swan pipes” at WWT sites, and since 1988, they have also been caught while flightless during the annual moult in Iceland. Captured birds were aged by their plumage characteristics as juveniles, immatures, or adults; adults of unknown age were assumed to be 2 y old at capture. Birds were then sexed by cloacal examination and fitted with a plastic leg-ring engraved with a unique 3-digit code, along with either British Trust for Ornithology or Icelandic Bird Ringing Scheme metal rings (65). Individually marked swans were identified on the wintering grounds throughout the course of the study by a network of both experienced professional and amateur ornithologists reading the rings through high-quality telescopes. Location, habitat, associated birds, and the number of juveniles were also recorded. Adults and juveniles were defined as associating if they moved in synchrony with the focal bird. If multiple resightings differed in the number of juveniles recorded for any particular season (starting in July and ending the next July), the mean was taken and rounded to the nearest integer. These data were used to assign individuals to two categories based on whether they had (1) or had not (0) successfully bred in that season (i.e., a binominal variable for breeding success). All data were first checked for quality and accuracy before being entered into a central database held by the WWT. A total of 223,039 observations of marked swans were recorded, including initial capture and subsequent recapture events, resighting, and dead recaptures. The vast majority (99.9%) of these observations were made in the United Kingdom, the Republic of Ireland, and Iceland.

### Extracting Winter Site Usage and Movement from the Database.

We only considered site usage and movement during the core winter period from October to March. This reduced the number of observations to 207,391 at 2,165 sites. For all seasons that an individual was resighted, we extracted the following information from the database: i) the number of times the bird was resighted, ii) the number of separate sites utilized, iii) the duration of the stay at each site, iv) the maximum duration at any one site which we then defined as being the main wintering site, v) whether the main site was a NR or not, v) the distance moved between sites, and vi) date of departure to the breeding grounds. Date of departure was defined as the last time a bird was seen in the United Kingdom or the Republic of Ireland between March and May. This window was based on GPS tracks from 50 individuals tracked on their migration to Iceland in 2008 and 2009 (35, 66).

We further constrained the database, only including records in which we had three consecutive years of data for any given individual, allowing us to isolate individuals where we could quantify breeding success in year *t* and determine winter site usage (NRs vs. non-NRs) in years *t*−1 and *t*−2 (*SI Appendix*, Fig. S3). Having data on two winters prior to an assessment of breeding success enabled us to identify individuals which had used NRs or non-NR areas in both previous winters. Importantly, it also allowed us to identify individuals which had moved out of, or into NRs in consecutive winters, and investigate the impact of this switch. Hence, NR usage is defined by an individual’s winter site use history over the previous two winters. Individuals using NRs in both previous winters are “within NR,” while those observed outside of NRs in both winters are “outside NR.” Individuals switching from NRs to other winter sites are defined as moving “out of NR,” while those moving into NRs are defined as moving “Into NR.” The final dataset consisted of 6,237 observations of 2,249 individual swans for which we had information on their breeding success over 29 seasons. All database extractions and data manipulations were carried out in the R programming language and environment for statistical computing (67).

### Survival Models.

Encounter histories (*n* = 6,950) were extracted from the database and utilized in the construction of Bayesian multistate survival models. We estimated survival (*ϕ*), encounter (or resighting) (*ρ*), and movement (or transition) (*ψ*) probabilities using multistate mark-recapture models (68, 69). To construct our multistate model, we considered two levels that defined winter site usage: within NR and outside NR and three age classes: “juvenile” (0 to 1 y), “immature” (1 to 2 y), and “adult” (2 y or greater) (*SI Appendix*, Fig. S4). Prior to the survival analysis, we conducted goodness-of-fit tests using U-CARE (v. 2.2) (70); this revealed evidence of trap dependence, and we thus considered different encounter probabilities for trap-aware and trap-unaware adults (71). By combining age, site, and the state “trap aware/unaware,” we defined 11 true (“alive,” “dead”) states and 7 observed (“encountered,” “not encountered”) states in our models. Because we were primarily interested in the long-term demographic effects of NRs, and the movement of individuals between them, we only fit a model with constant survival, encounter, and movement probabilities for each age class. All models were fitted within a Bayesian framework via Markov Chain Monte Carlo (MCMC) in JAGS (Just Another Gibbs Sampler, v. 4.3.0) (72) using the “jagsUI” library (v. 1.5.0) (73) in R (v. 3.5.0; R Core Team 2018). We specified vague priors of *U* (0,1) for all model parameters and ran three independent MCMC chains of 15,000 iterations with a burn-in of 5,000 iterations and thinning to every 10 posterior samples. To check for MCMC convergence, we computed the Brooks–Gelman–Rubin statistic ( $\hat{R}$ ) (74). In our results, $\hat{R}$ values were below 1.02 for all parameters, indicating no evidence of lack of convergence.

### Productivity Model.

We constructed generalized linear mixed models with a binomial error structure (and a logit link), with cygnets (0/1) as the dependent variable. The maximal model contained all terms which we believed would be likely to affect the probability of breeding and were extractable from the database. The model included both age and the quadratic term for age as fixed effects because we expected the relationship between age and breeding success to be nonlinear. We also included a fixed effect describing each individual’s breeding experience (quantified as the total number of cygnets previously produced), as the probability of breeding can increase with experience in long-lived species (75).

We used resighting data to determine whether the main site used in the previous two winters was a NR or not (see above). Swans were often resighted at multiple sites during each winter; therefore, we took the site at which individuals spend the most time as being their main wintering site. Individuals using NRs as their main site in both winters were defined as being within a NR, while those not using NRs were defined as being outside of a NR. Those who switched from NRs to non-NR sites (or vice versa) between the two winters were defined as either moving into NRs or moving out of NRs (*SI Appendix*, Fig. S3).

As population breeding success is variable between years, we also attempted to incorporate a measure of population breeding success into the model. This, however, led to model convergence problems and so was dropped from the final model. We included season and individual swan identification coded as crossed random effects. In addition, we used a subset of the data to examine any potential effects of density dependence. The model was restricted to observations where the previous season’s main site (winter *t*−1, *SI Appendix*, Fig. S1) was a NR. We used maximum swan count data as a proxy for density and incorporated this within the model as a covariate (*SI Appendix*, Table S2*C*). This model was based on 4,420 observations of 1,561 individual swans over 28 seasons.

All predictors were standardized to ensure they were on a common scale, which increases the interpretability of the parameter estimates particularly when interactions are involved (76). Further details on the frequentist models and model selection can be found in *SI Appendix* (*SI Appendix*, Table S2). Models were fitted using the “lme4” package (77) and the “bobyqa” optimizer.

To adequately propagate the uncertainty around our breeding probabilities into our demographic model (see below), we constructed the minimally adequate model from the frequentist model selection (above) in a Bayesian framework. We assume that the response variable ( y ) follows a Bernoulli distribution with probability α , which was modeled as linear functions of the explanatory variables using a logit link:

[1] $$\begin{array}{c} y_{l,m,k}\sim \mathrm{Bernoulli}\left(\alpha_{l,m,k}\right),\\ \mathrm{logit}(\alpha_{l,m,k})=\beta_{1}+\beta_{2}x_{l}+\beta_{3}z_{k}+\beta_{4}x_{l}z_{k}^{2}+\beta_{5}w_{k}+b_{m}+b_{k},\\ \;l=1,\ldots ,4,\;\;\;m=1,\ldots ,29,\;\;\;k=1,\ldots ,n_{l,m}=2,249,\\ \;b_{m}\sim N\left(0,\sigma_{1}^{2}\right),\;\;\;\;b_{k}\sim N\left(0,\sigma_{2}^{2}\right), \end{array}\;$$

where $y_{l,m,k}$ are the individual observations of whether an individual swan ( k ) had cygnets or not (1 or 0), in season m at a site with protection status l ; the β’s are coefficients to be estimated for the fixed-effects; $b_{m}$ denotes the season-level random effect and $b_{k}$ the individual-level random effect; $x_{l}$ is a covariate vector denoting protection status; $z_{k}$ is a covariate vector denoting the age of each swan; and $w_{k}$ is a covariate vector denoting the previous breeding experience of each swan.

In addition, we used a vector of ages between 1 and 27 y old and random draws from a Poisson distribution P(*r*), where *r* (rate) = the mean previous breeding experience in the dataset, bounded between 0 and the corresponding entry in the age vector to produce a posterior sample for previous breeding experience for each MCMC iteration, such that a swan of 25 y old could have between 0 and 24 y of previous breeding experience (but not 26 y). We then used these posterior samples to produce predicted posterior breeding probabilities by age for each of the four protection status groups (Fig. 2).

The model was implemented using MCMC estimation in JAGS (v. 4.3.1) via the jagsUI library (v. 1.5.0) for program R (v. 3.5.0). We specified vague priors: $N\left(0,,,10^{-3}\right)$ for regression coefficients (where 10^−3^ is precision) and U (0,5) for SDs ( σ ), with the precision specified as $\sigma^{-2}$ . We ran three independent MCMC chains of 550,000 iterations with 50,000 iterations as burn-in, thinning to every five posterior samples, and checked for convergence using Brooks–Gelman–Rubin statistics (all $\hat{R}$ values < 1.01).

### Demographic Models.

We used a population projection model with demographic stochasticity and parameter uncertainty to model the changes in the Whooper swan population size within NRs and outside NRs between 1985 and 2030 (Fig. 3). The model approximates a Leslie matrix model with 20 age classes (1 juvenile, 1 immature, and 18 breeding adult classes), a postbreeding census, and where all individuals mature at age 2, but survival ( ϕ ), movement ( ψ ), and fecundity ( F ) rates were all stochastic using observed or modeled means ± SDs. For the fecundity ( F ) component of the model:

[2] $$F={\hat{y}}_{age}\times f\times R\times \phi_{a},$$

where ${\hat{y}}_{age}$ is the modeled age-specific breeding probability (from Eq. **1**), f is the proportion of females in the breeding population (taken to be 0.5), $\phi_{a}$ is adult survival, and R is the mean brood size (number of offspring per female). The age-specific breeding probability ( ${\hat{\alpha }}_{age}$ ) was randomly sampled from a beta distribution of the form:

[3] $$\begin{array}{c} \;\mathrm{Beta}\left(\alpha_{age,l},\beta_{age,l}\right),\\ l=1,\ldots ,4,\;\;age=1,\ldots ,20, \end{array}$$

where the parameters of a beta distribution were derived using the posterior mean ( $\bar{x}$ ) and standard deviation (sd ) for the relevant age ( age ) and site status ( l ) (*SI Appendix*, Table S1). That is, $\alpha_{age,l}={\bar{x}}_{age,l}\times u_{age,l}$ , $\beta_{age,l}={(1-\bar{x}}_{age,l})u_{age,l}$ and $u_{age,l}=\left(\frac{{\bar{x}}_{age,l}\times \left(1-{\bar{x}}_{age,l}\right)}{sd_{age,l}^{2}}\right)-1$ . We used the same approach to randomly sample juvenile ( $\phi_{j}$ ), immature ( $\phi_{i}$ ), and adult survival ( $\phi_{a}$ ) and juvenile ( $\psi_{j}$ ), immature ( $\psi_{i}$ ), and adult movement ( $\psi_{a}$ ) probabilities from a Beta distribution, with all 18 breeding adult classes using the same mean and SD (values in Fig. 1 and main text). Similarly, we used the mean ( $\bar{x}$ = 2.50) and SD ( sd = 0.483) of annual brood size data from the WWT’s Swan Monitoring Program for the years 1990 to 2011 and randomly sampled R from a truncated Gamma distribution: $Gamma\left(\vartheta ,\omega \right)$ , where ϑ = the shape parameter ( $\vartheta ={\bar{x}}^{2}/{\mathrm{sd}}^{2}$ ), and ω = the scale parameter ( $\omega =\bar{x}/{\mathrm{sd}}^{2}$ ), with the distribution truncated at the minimum (1.5) and maximum (3.6) annual mean brood size observed.

To seed our Bayesian model, we took the dominant eigenvector of a corresponding deterministic, 20 age-class Leslie matrix model and multiplied this by population census data for the British Isles for swans in WWT sites, or NRs (776 individuals), and those outside NRs (15,924 individuals) in 1985 to obtain starting stable age distributions. We then ran the stochastic model once for 46 y, took the posterior mean of the number of individuals in each age class at the end of the model run as the stochastic stable age distribution, and reseeded the model with these values as starting populations. We then ran the models used for inference, using three MCMC chains of 50,000 samples, a burn-in of 10,000, and thinning to every 10th posterior sample. We used beta prior distributions for all stochastic survival and movement probabilities and Poisson distributions to map the number of individuals in each of the 20 states from year *t* to *t* + 1 (thus allowing for demographic stochasticity). The model was run for *t* = 46 y (thus, simulating the population trajectory from 1985 to 2030) and the population growth rate ( λ ) was calculated as the posterior mean of all the 45 annual growth rates:

[4] $$\lambda =\frac{\left(\sum_{t=1}^{45}\left(n_{t+1}/n_{t}\right)\right)}{45}.$$

We ran one model scenario in which swans were allowed to transition between NRs and non-NRs, then breed appropriately, and compared the modeled population trajectory for 1985 to 2014 to the observed population counts (Fig. 3 *A* and *B*). We then ran a second scenario seeded with the population outside NRs and with no movement probabilities, to simulate a situation where the NRs had never been set up, again seeding this model following the approach above with a starting stable age distribution based on the 15,924 individuals outside WWT sites (NRs) in 1985 and projecting to 2030 (Fig. 3*B*). We compared the mean (±80% credible intervals, CIs) between these two scenarios for 2020, 2025, and 2030 (Fig. 3*C*). Finally, to estimate the effect of a proportional change in the vital rates on population growth, we recalculated λ after reducing individual vital rates, one at a time by 5%, and calculated the elasticity ( E ) of the survival and breeding probabilities as:

[5] $$E=\frac{\left|\left(\lambda -\lambda '\right)/\lambda \right|}{\left|\left(r-r'\right)/r\right|},$$

where r is the value of the vital rate of interest (i.e., $\phi_{j},\phi_{i},\phi_{a}$ or $\hat{\alpha }$ ) (78).

## Supplementary Material

Appendix 01 (PDF)

## Data Availability

All data and code needed to evaluate the conclusions in this paper are freely available on GitHub (https://github.com/rbsherley/Swans) and have been archived in a scientific repository, Zenodo, with the following DOI: 10.5281/zenodo.7454145 (79).
