# Supplementary material for: Demographic rates reveal the benefits of protected areas in a long-lived migratory bird
Source: Proc Natl Acad Sci U S A. 2023 Mar 13;120(12):e2212035120. doi: 10.1073/pnas.2212035120 (PMC10041063; doi:10.1073/pnas.2212035120)
Supplement: Supplementary file 1 — Appendix 01 (PDF) [file pnas.2212035120.sapp.pdf]

Supplementary Materials for Soriano-Redondo et al. Demographic rates reveal the benefits of protected areas in a long-lived migratory bird

Productivity Models

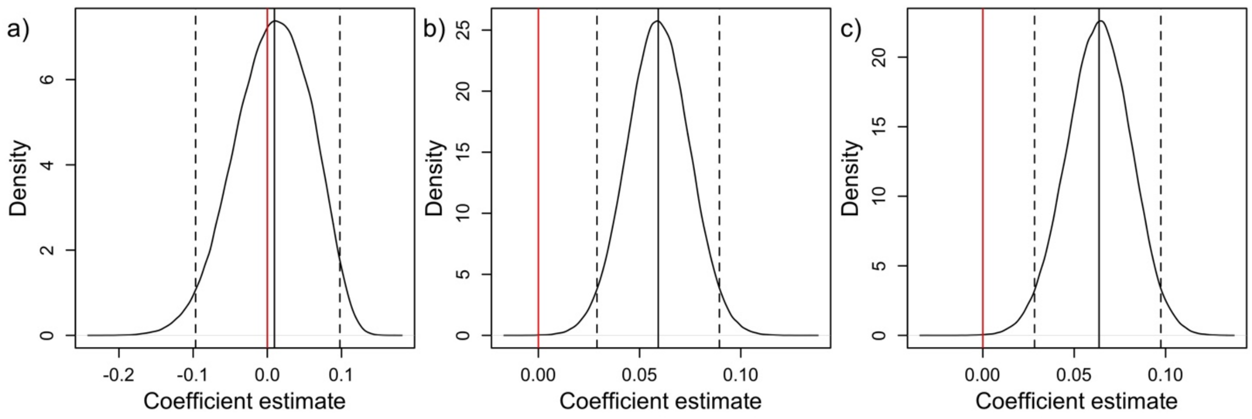

Figure S1. Posterior probability distributions for the estimated effect of the winter site usage and age interaction from the minimal model (eqn. 1) used to estimate age-specific Whooper Swan breeding probabilities for birds Within Nature Reserves (NRs), Outside NRs, moving into NRs and moving Out of NRs. Density plots show the difference between birds Within NRs and a) those moving Into NRs; b) Outside NRs; and c) those moving Out of NRs, with the mean (solid black line) and 95% credible intervals (dashed lines) shown. Values above zero (red line) indicate a positive effect size while those below zero indicate a negative effect size.

Table S1. Mean (standard deviation) for age-specific breeding probabilities for Whooper Swans Within Nature Reserves (NR), Outside NR, moving Into NR and moving Out of NR, estimated from the generalized linear mixed model described in equation 1 and used (up to the age of 20) in the population projection model.

Age (years)	Within NR	Outside NR	Into NR	Out of NR
1	0.037 (0.011)	0.067 (0.020)	0.039 (0.017)	0.047 (0.015)
2	0.088 (0.028)	0.132 (0.042)	0.088 (0.035)	0.093 (0.032)
3	0.172 (0.061)	0.224 (0.077)	0.171 (0.069)	0.162 (0.061)
4	0.278 (0.106)	0.329 (0.119)	0.273 (0.115)	0.249 (0.101)
5	0.374 (0.148)	0.423 (0.157)	0.367 (0.156)	0.334 (0.143)
6	0.437 (0.180)	0.49 (0.185)	0.430 (0.186)	0.401 (0.177)
7	0.463 (0.202)	0.528 (0.204)	0.456 (0.206)	0.442 (0.201)
8	0.452 (0.216)	0.539 (0.217)	0.449 (0.220)	0.457 (0.218)
9	0.412 (0.222)	0.529 (0.225)	0.413 (0.228)	0.450 (0.227)
10	0.348 (0.219)	0.501 (0.231)	0.355 (0.230)	0.427 (0.231)
11	0.269 (0.203)	0.459 (0.235)	0.287 (0.225)	0.390 (0.231)
12	0.187 (0.172)	0.404 (0.233)	0.218 (0.210)	0.342 (0.225)
13	0.115 (0.131)	0.341 (0.226)	0.159 (0.189)	0.289 (0.215)
14	0.062 (0.008)	0.275 (0.211)	0.112 (0.165)	0.234 (0.199)
15	0.029 (0.051)	0.210 (0.190)	0.077 (0.141)	0.180 (0.178)
16	0.012 (0.026)	0.151 (0.161)	0.053 (0.118)	0.132 (0.153)
17	0.004 (0.011)	0.102 (0.131)	0.037 (0.101)	0.093 (0.127)
18	0.001 (0.004)	0.064 (0.099)	0.026 (0.085)	0.061 (0.100)
19	<0.001 (0.001)	0.039 (0.072)	0.018 (0.072)	0.039 (0.077)
20	<0.001 (<0.001)	0.022 (0.05)	0.013 (0.061)	0.024 (0.056)
21	<0.001 (<0.001)	0.012 (0.033)	0.010 (0.054)	0.014 (0.041)
22	<0.001 (<0.001)	0.006 (0.021)	0.007 (0.047)	0.008 (0.030)
23	<0.001 (<0.001)	0.003 (0.013)	0.005 (0.042)	0.005 (0.021)
24	<0.001 (<0.001)	0.001 (0.008)	0.004 (0.037)	0.003 (0.015)
25	<0.001 (<0.001)	0.001 (0.006)	0.003 (0.035)	0.001 (0.010)
26	<0.001 (<0.001)	<0.001 (0.003	0.003 (0.031)	0.001 (0.009)
27	<0.001 (<0.001)	<0.001 (0.002	0.002 (0.029)	<0.001 (0.006)

Frequentist models

Table S2. Frequentist productivity models. (a) Model with both terms; Previous Breeding Success and Population Breeding Success. This model produced singularity errors, therefore as Previous Breeding success had a much larger parameter estimate and significance we removed the term Population Breeding Success from the final model (b). Note that the explanatory power of the model is only slightly higher in the final model. This final model was then used to construct the Bayesian model. In addition, we produced a model to look at any potential density dependent effects utilising swan count data from nature reserve sites as a proxy for density (c). The model only contained data for individuals which has spent the previous season at a nature reserve, hence the factor which defines winter site usage was restricted to 2 levels (Within NR and Out of NR).

(a) Previous Breeding Success & Population Breeding Success

	Estimate	Standard Error	z-value	p-value	Significance
Age	0.219	0.074	4.032	<0.001	***
Age^2	-0.554	0.051	-10.783	<0.001	***
Into NR	-0.039	0.282	-0.139	0.889	
Outside NR	0.238	0.137	1.729	0.083	
Out of NR	-0.169	0.177	-0.954	0.340	
Previous Breeding Success	1.657	0.068	24.443	<0.001	***
Population Breeding Success	-0.069	0.032	-2.122	0.034	*
Into NR * Age	0.072	0.253	0.286	0.775	
Outside NR * Age	0.248	0.064	3.845	<0.001	***
Out of NR * Age	0.268	0.073	3.680	<0.001	***

$R^2_{(m)}$ = 0.42, $R^2_{(c)}$ = 0.54. The base level for the NR factor was Within NR.

(b) Previous Breeding Success Only

	Estimate	Standard Error	z-value	p-value	Significance
Age	0.295	0.074	3.974	<0.001	***
Age^2	-0.553	0.051	-10.769	<0.001	***
Into NR	-0.042	0.282	-0.150	0.881	
Outside NR	0.224	0.138	1.633	0.102	
Out of NR	-0.189	0.178	-1.062	0.288	
Previous Breeding Success	1.660	0.068	24.473	<0.001	***
Into NR * Age	0.072	0.253	0.283	0.777	
Outside NR * Age	0.248	0.064	3.854	<0.001	***
Out of NR * Age	0.268	0.073	3.689	<0.001	***

$R^2_{(m)}$ = 0.40, $R^2_{(c)}$ = 0.54. The base level for the NR factor was Within NR.

(c) Density Dependence Model

	Estimate	Standard Error	z-value	p-value	Significance
Age	0.247	0.090	2.740	<0.006	**
Age^2	-0.502	0.053	-9.392	<0.001	***
Out of NR	-0.229	0.191	-1.202	0.230	
Previous Breeding Success	1.570	0.079	19.799	<0.001	***
Density	-0.151	0.178	-0.847	0.397	
Out of NR * Age	0.252	0.072	3.532	<0.001	***

$R^2_{(m)}$ = 0.38, $R^2_{(c)}$ = 0.54. The base level for the NR factor was Within NR.

Departure date GPS data.

Data from 55 GPS tagged swans were using to explore differences in departure from wintering sites (*n* = 55), departure from the UK / Ireland (*n* = 47), and arrival in Iceland (*n* = 48), between swans which had wintered primarily at nature reserves and those wintering outside nature reserves. Swans were tagged in three years (2008 *n* = 10, 2009 *n* = 40 & 2020 *n* =5). GPS tracks were recorded from three nature reserve sites (Welney *n* = 16, Martin Mere *n* = 20 & Caerlaverock *n* = 6), and three non-nature reserve sites (County Mayo *n* =1, Ireland, Country Galway, Ireland *n* = 1 & Lough Beg, UK *n* =11).

As the data did not met the assumptions from ANOVA we carried out Kruskal-Wallis test using the Day number (January 1st = 1) as the dependant variable and whether individuals wintered at a nature reserve or not as a 2 level grouping variable (Outside NR & Within NR).

We found no significant differences in departure from the winter site (mean Outside NR = 83.23, mean Inside NR = 82.29, χ^2 = 0.077 p = 0.781), departure from the UK / Ireland (mean Outside NR = 84.36, mean Inside NR = 90.44, χ^2 = 3.628 p = 0.057), or arrival in Iceland (mean Outside NR = 88.5, mean Inside NR = 91.75, χ^2 = 2.560 p = 0.110), between swans wintering within nature reserves and wintering outside of nature reserves.

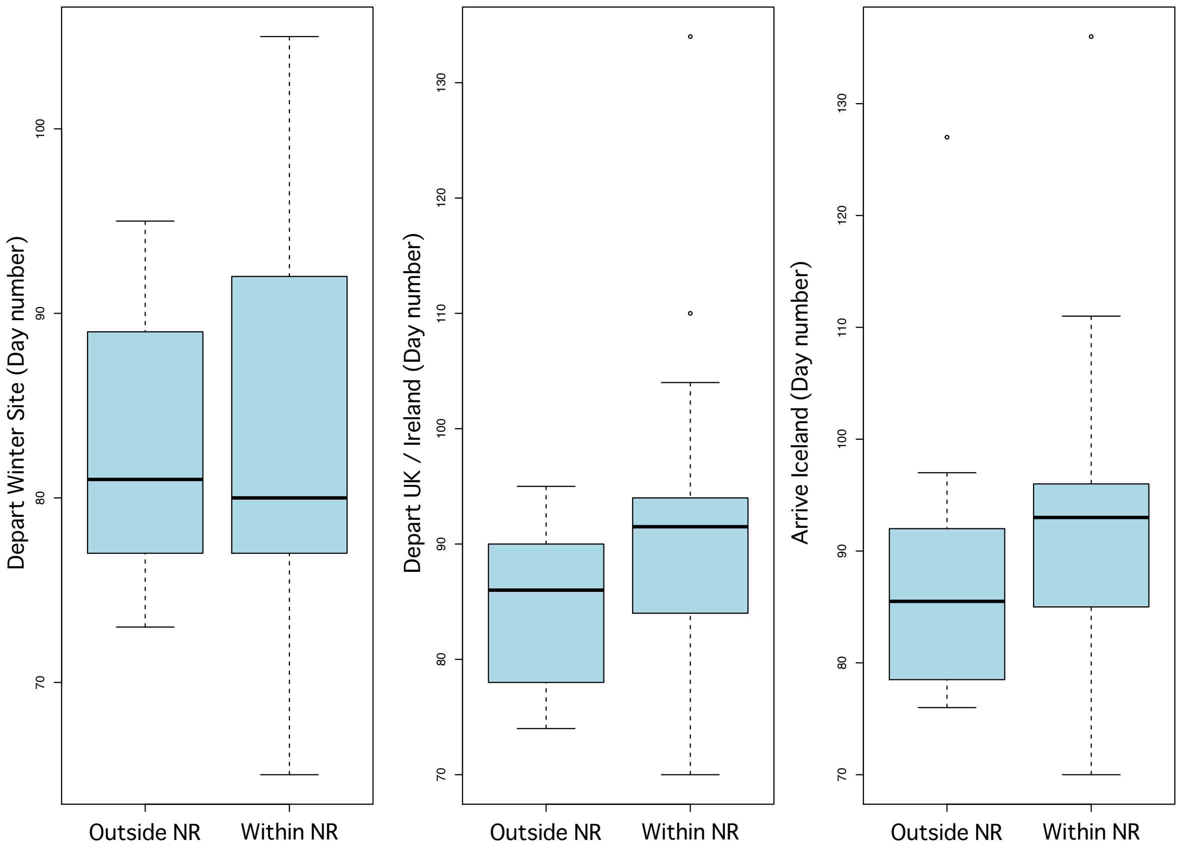

Figure S2. Boxplots of departure date (as day number) from the wintering site, departure from UK or Ireland, and arrival to Iceland as a function of wintering outside or within a nature reserve.

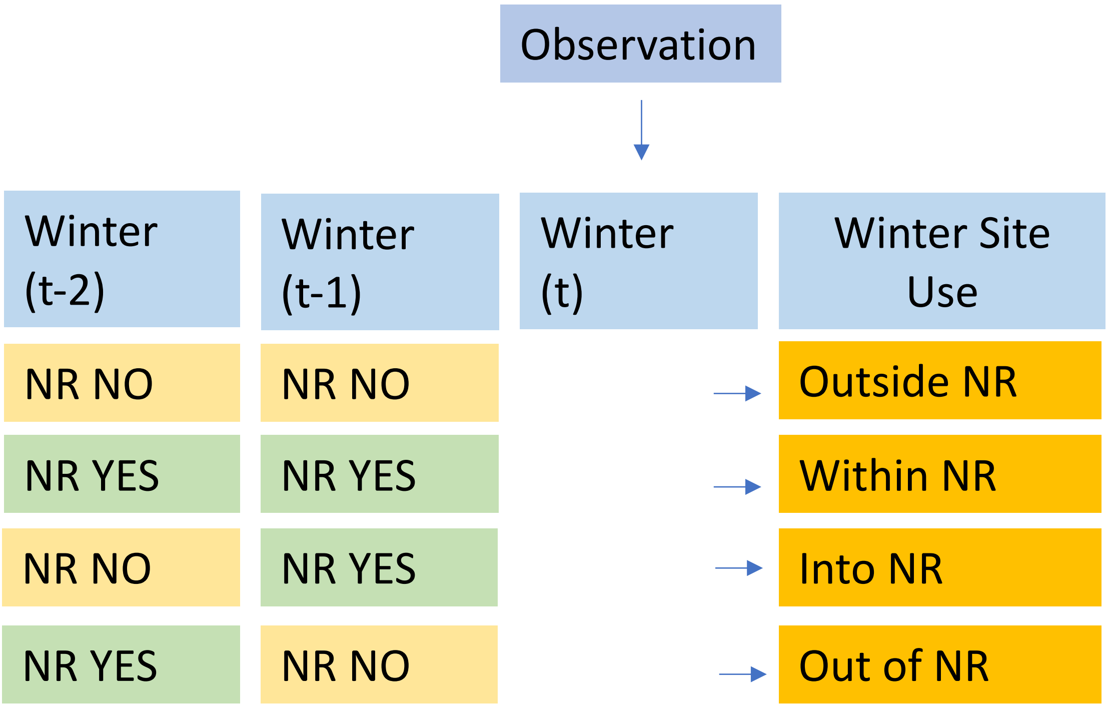

Figure S3. Conceptual representation of the classification of the wintering site use depending on the wintering grounds used in the previous winters.

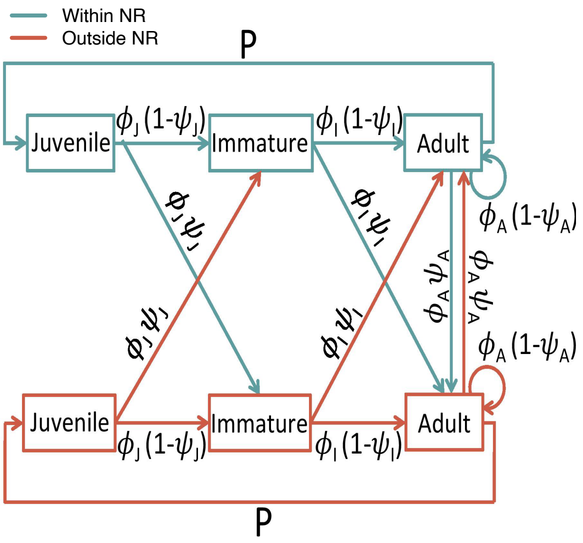

Figure S4. Graphic representation of the parametrization of the population projection model based on juvenile (J), immature (I) and adult (A) survival (φ) and movement (ψ) rates.
